# Melamine–Glyoxal–Glutaraldehyde Wood Panel Adhesives without Formaldehyde

**DOI:** 10.3390/polym10010022

**Published:** 2017-12-24

**Authors:** Xuedong Xi, Antonio Pizzi, Siham Amirou

**Affiliations:** LERMAB, University of Lorraine, 27 rue Philippe Seguin, 88000 Epinal, France; xuedong.xi@univ-lorraine.fr (X.X.); siham.amirou@univ-lorraine.fr (S.A.)

**Keywords:** ionic liquids, melamine resins, wood adhesives, plywood, ionic liquids role, MALDI-ToF, FTIR

## Abstract

(MGG’) resin adhesives for bonding wood panels were prepared by a single step procedure, namely reacting melamine with glyoxal and simultaneously with a much smaller proportion of glutaraldehyde. No formaldehyde was used. The inherent slow hardening of this resin was overcome by the addition of *N*-methyl-2-pyrrolidone hydrogen sulphate ionic liquid as the adhesive hardener in the glue mix. The plywood strength results obtained were comparable with those obtained with melamine–formaldehyde resins pressed under the same conditions. Matrix assisted laser desorption ionisation time of flight (MALDI ToF) and Fourier transform Infrared (FTIR) analysis allowed the identification of the main oligomer species obtained and of the different types of linkages formed, as well as to indicate the multifaceted role of the ionic liquid. These resins are proposed as a suitable substitute for equivalent formaldehyde-based resins.

## 1. Introduction

Melamine–formaldehyde and melamine–urea–formaldehyde resins and adhesives are extensively used in particular for impregnated paper surface overlays and for plywood and particleboard panel binders [1]. The problem with these resins is now the presence of formaldehyde and its emission, as this chemical has been reclassified to carcinogenic category 1B and mutagen category 2 according to the “Classification, Labelling and Packaging of substances and mixtures” (CLP) of the EU Regulations.

Glyoxal has been chosen to substitute formaldehyde in some of these resins due to its capacity for giving clear adhesive resins and thus presenting the same appearance as UF-, MF- and MUF-bonded wood panels.

Glyoxal is nontoxic (LD50 rat ≥ 2960 mg/kg; LD50 mouse ≥ 1280 mg/kg when compared to formaldehyde’s LD50 of 75 mg/kg) and non-volatile in the conditions in which formaldehyde is volatile when used in panels bonded with formaldehyde-based adhesives [2,3]. Because of advantages such as its mature production technology, low cost and easy biodegradation, glyoxal (G) has been widely used as a green environmental additive in the papermaking and textile industries [4]. In wood adhesive, it is mainly used to substitute formaldehyde (F) partially or totally, in applications as a crosslinking agent or curing agent in natural wood adhesives such as tannin-based adhesives [5,6], lignin-based adhesives [7] and protein-based adhesives [8]. While glyoxal is nontoxic and non-volatile it is also much less reactive than formaldehyde and the presence of two vicinal aldehyde groups partially, but only partially, offsets this drawback. Moreover, the literature about glyoxal-based resins application for wood adhesives and resins is rather scarce.

Recently, much work has been conducted to prepare and study the reaction mechanism and structure of environmentally friendly urea-based aminoresins by choosing glyoxal (G) to substitute formaldehyde (F) partially or totally so as to eliminate or markedly decrease formaldehyde (F) emission [9,10,11,12]. Urea–glyoxal (UG) and urea–glyoxal–formaldehyde (UGF) resins have been successfully synthesized and used as wood adhesive for plywood, interior decoration and furniture material [10,11] as well as for particleboard [12]. Equally, melamine–glyoxal resins have been formulated and while they were capable of yielding reasonable bond strength to satisfy relevant plywood standards, they did not yield internal bond (IB) strength results for particleboard to the level needed to satisfy relevant standards [13]. This was shown to be due to the energy of activation of cross-linking of the melamine-glyoxal (MG) resins being higher than formelamine-formaldehyde ( MF) resins [13]. Acceptable gel times are obtainable only at temperatures higher than 150 °C, thus too high to be able to properly cure the resin in a particleboard core where the max temperature reached is of 110–120 °C. This rendered possible some applications such as MG resins impregnated paper surface overlays were the resin is in direct contact with the hot platen of the press at 180 °C but showed such resins limitations as binders for wood particleboard [13].

Ionic liquids are salts in the liquid state. In general, the term is restricted to salts whose melting point is below some arbitrary temperature, such as 100 °C. They are salts in which the ions are poorly coordinated, this causing these solvents to be liquid below 100 °C. Ionic liquids are largely made of ions and short-lived ion pairs. Ionic liquids are becoming of considerable importance in many applied fields of technology [14,15,16]. The potential market of ionic liquids is large. Currently, ionic liquids have an estimated market value of 1.3 billion US dollars per year, mainly as replacement for traditional organic solvents in petrochemical and pharmaceutical industries [14]. Their market is expected to increase rapidly, this likely upsurge being attributed to the greener characteristics of these materials and good solubility in water and organic solvents [14].

Recently, the energy of activation of cross-linking of urea–glyoxal resins was effectively and markedly decreased by the use of ionic liquids (IL) as resin hardeners and additives [12,17]. This made it possible to obtain the same performance of UF resins by using UG + IL resins to even bond particleboard. The same approach has then been used in this article for melamine–glyoxal resins with the added variation of using a small proportion of glutaraldehyde (G’) further condensed with the MG resin to yield a melamine–glyoxal–glutaraldehyde (MGG’) adhesive presenting even better water resistance of the wood panels bonded with it.

## 2. Materials and Methods

### 2.1. Preparation of N-Methyl-2-Pyrrolidone Hydrogen Sulphate ([HNMP] [HSO_4_^−^]) Ionic Liquid

[HNMP] [HSO_4_^−^] ionic liquid was synthesized according to the method of Wang et al. [18]. 1.0 mol *N*-methyl pyrrolidone (LERMAB, Epinal, France was weighed and placed into a 250 mL three-neck flask. Then 1.0 mol sulphuric acid (Carlo Erba, Milan, Italy) was added dropwise into the 250 mL three-neck flask with a dropping funnel under continuous mechanical stirring at room temperature. A considerable amount of white smoke appeared during the dropwise addition of acid. The mixture became a sticky, light yellow transparent liquid after stirring at 80 °C for 4 h. The resultant liquid *N*-methyl-2-pyrrolydone hydrogen sulphate ([HNMP] [HSO_4_^−^]) was washed three times with ether and ethyl acetate, respectively, then it was dried under vacuum to remove the ether and ethyl acetate.

### 2.2. Preparation of MG and MGG’ Resins

The MG resins were prepared at M/G molar ratio: 1:6, according to the method of Deng et al. [13]. Glyoxal (40% water solution, Sigma-Aldrich, St.Louis, MO, USA) was placed in the three-neck flask and the pH was adjusted to 4.5 using 33% NaOH. Subsequently, melamine was added, and the mixture was heated to 60 °C for 1 h. The reaction mixture was cooled to room temperature ready for use.

Under the same reaction conditions, different parts of glyoxal were substituted with glutaradehyde (50% water solution, Sigma Aldrich, St. Louis, MO, USA) to prepare MGG’ resins. The relative molar proportions of glyoxal to glutaraldehyde are shown in Table 1 and Table 2.

### 2.3. Fourier Transform Infrared Spectrometry (FTIR)

To confirm the presence of relevant structures, a Fourier Transform Infra Red (FTIR) analysis was carried out using a Shimadzu IRAffinity-1 spectrophotometer (Kyoto, Japan). A blank sample tablet of potassium bromide, ACS reagent from ACROS Organics (Acros Otganics, Geel, Belgium), was prepared for the reference spectrum. A similar tablet was prepared by mixing potassium bromide with 5% *w/w* of the sample powders to be analyzed. The spectrum was obtained in transmission measurement by combining 32 scans with a resolution of 2.0 (Perkin-Elmer, Villebon, France).

### 2.4. Matrix Assisted Laser Desorption Ionisation Time of Flight (MALDI-ToF) Spectrometry

Samples for Matrix assisted laser desorption ionization time-of-flight (MALDI-ToF, AXIMA Performance, Shimadzu, Manchester, UK) analysis were prepared first dissolving 5 mg of sample powder in 1 mL of a 50:50 *v/v* acetone/water solution. Then 10 mg of this solution is added to 10 µL of a 2,5-dihydroxy benzoic acid (DHB) matrix. The locations dedicated to the samples on the analysis plaque were first covered with 2 µL of a NaCl solution 0.1 M in 2:1 *v/v* methanol/water, and predried. Then 1 µL of the sample solution was placed on its dedicated location and the plaque is dried again. MALDI-ToF spectra were obtained using an Axima-Performance mass spectrometer from Shimadzu Biotech (Kratos Analytical Shimadzu Europe Ltd., Manchester, UK) using a linear polarity-positive tuning mode. The measurements were carried out making 1000 profiles per sample with two shots accumulated per profile. The spectrum precision is of +1 Da.

### 2.5. Plywood Preparation and Testing

The performance of the MGG’ resins was tested by preparing laboratory plywood panels and evaluating their shear strength dry, after 24 h cold water soaking, and after 2 h in boiling water, tested wet. Triplicate three-ply laboratory plywood panels of 450 mm × 300 mm × 5 mm were prepared for each MGG’ adhesive resin and 2 mm poplar (*Populus tremuloides*) veneers. To the glue mixes was added 10% by weight [HNMP] [HSO_4_^−^] ionic liquid as hardener on total MGG’ resin solids. The glue spread used was of 260 g/m^2^ double glue line, and hot pressing time was of 6 min at 150 °C and 1.5 MPa pressure. After hot pressing, the plywood was stored under ambient conditions (20 °C and 12% moisture content) for 48 h before testing according to China National Standard GB/T 14074 (2006) [19] and China National Standard GB/T17657 (1999) [20] which require a minimum average shear strength of 0.7 MPa on five specimens tested, and European Norm EN 636:2012 (2012) [21].

## 3. Results and Discussion

**Scheme 1:**
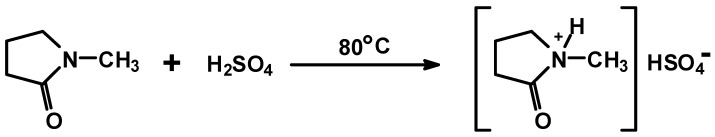
Preparation of *N*-methyl-2-pyrrolidone hydrogen sulphate ([HNMP] [HSO_4_^−^]).

The same principle of using an ionic liquid as a hardener that has been used for urea–glyoxal resins [12,17] was applied to melamine–glyoxal (MG) and melamine–glyoxal–glutaraldehyde (MGG’) resins for which, *N*-methyl-2-pyrrolidone hydrogen sulphate ([HNMP] [HSO_4_^−^]) was used as hardener. As this material is relatively expensive it was prepared in the laboratory according to the method of Wang et al. [15] and used with a minimum of purification [12,17] (Scheme 1).

The MG resin evolved into the MGG’ resins given the better results that were obtained with such a combination. The double reaction, in two successive steps of melamine with glyoxal and glutaraldehyde has led to clear resin with no formaldehyde, each presenting distinct characteristics. These are shown in Table 1 which also reports the strength results of laboratory plywood panels prepared by using the different melamine–glyoxal–glutaraldehyde resins to which had been added 10% of [HNMP] [HSO_4_^−^] ionic liquid. These satisfy the relevant requirements of international standards for both dry strength and strength after 24 h cold water soaking. In the two best cases they also satisfy the requirements for exterior grade bonds as their strength is still acceptable after the boiling water test.

### 3.1. Adhesives Bonding Performance

The first characteristic that can be noticed from Table 2 is that the proportion of glutaraldehyde increases the viscosity, and more interestingly the dry and wet tensile strengths increase up to a proportion of 20% molar of glutaraldehyde. A higher proportion nonetheless causes the viscosity to increase to such an extent that the resin solidifies in the reactor. Equally remarkable is the finding (Table 2) that the tensile strength increases after 24 h cold water soak. The effect is very marked and becomes more evident as the proportion of glutaraldehyde increases. At first it was thought that the water repellent –(–CH_2_–)_3_– chain of glutaraldehyde caused the effect, but while this contributes to maintaining the dry strength once in water it does not justify the marked increase in strength noticed. It does however, contribute also to the resistance of the bond to boiling water as the bond performance improves as the relative proportion of glutaraldehyde increases.

**Figure 1:**
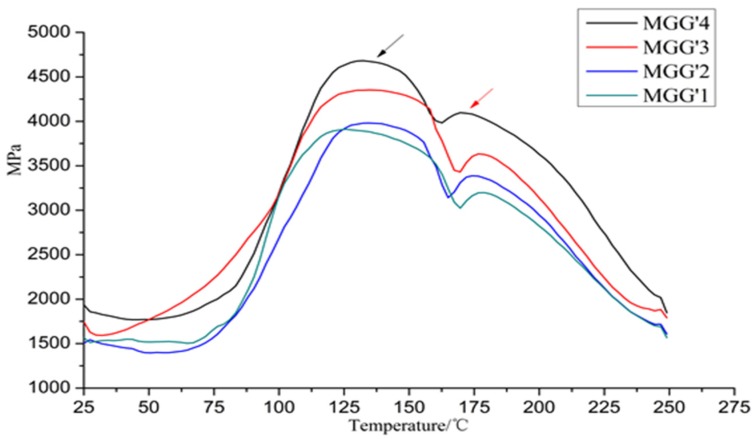
Thermomechanical analysis (TMA) curves showing the Modulus of Elasticity (MOE) as a function of temperature for the hardening of the MGG’ adhesives from Table 1 and Table 2.

Thermomechanical analysis experiments has shown that the hardening of the resin occurs in two phases (indicated with two arrows in Figure 1) a second increase in modulus occurring as the temperature, hence the curing time increases. This appeared to indicate that while the two aldehydes have been added simultaneously in the reactor, one is less reactive (glutaraldehyde) and had both reacted with the melamine in the reactor, one of them was contributing to cross-linking earlier than the other aldehyde. Considering the difference in reactivity of the two aldehydes when the MGG’ resins was prepared, this should indicate that a MG resin is formed on to which some glutaraldehyde is also eventually linked. Cross-linking then, will depend first from the more numerous, still reactive hydroxyethyl groups formed by the first addition of glyoxal onto melamine to form bridges. Second from the less reactive hydroxypentyl groups yielded by the grafting reaction of glutaraldehyde onto the MG resin. This would explain the two phases of hardening of the MGG’ resin seen in Figure 1. To show that this was the case, matrix assisted laser desorption ionisation time of fight (MALDI-ToF) mass spectrometry analysis of different resins and intermediates was carried out. This presented several points of interest: (i) first of all the composition and oligomers distribution in the MG and in particular the MGG’ resin; (ii) second, and of equal interest, was to find out what was the interaction of the ionic liquid with both the two aldehydes, with melamine, with the MGG’ resin so as to finally deduce its mechanism of action.

**Figure 2:**
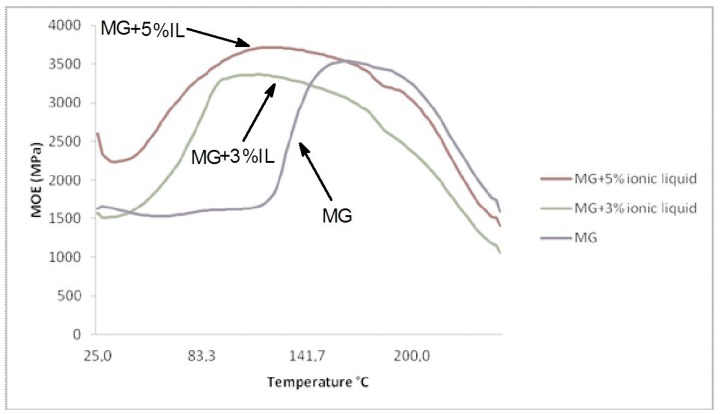
Comparative thermomechanical analysis curves of the rate of hardening of MG resins with and without addition of IL.

Thermomechanical analysis curves of the MG resin hardening in respect of the hardening of the MG resin + 3% IL and MG resin + 5%IL (Figure 2) shows clearly the rise at a much lower temperature of the MOE of the IL catalysed resins indicating their much faster curing. This confirms the role of IL in lowering of the energy of activation of condensation and hardening of MG resins.

### 3.2. Analysis of Chemical Species Formed in the Reactions Analysed by MALDI-ToF Spectrometry

**Figure 3:**
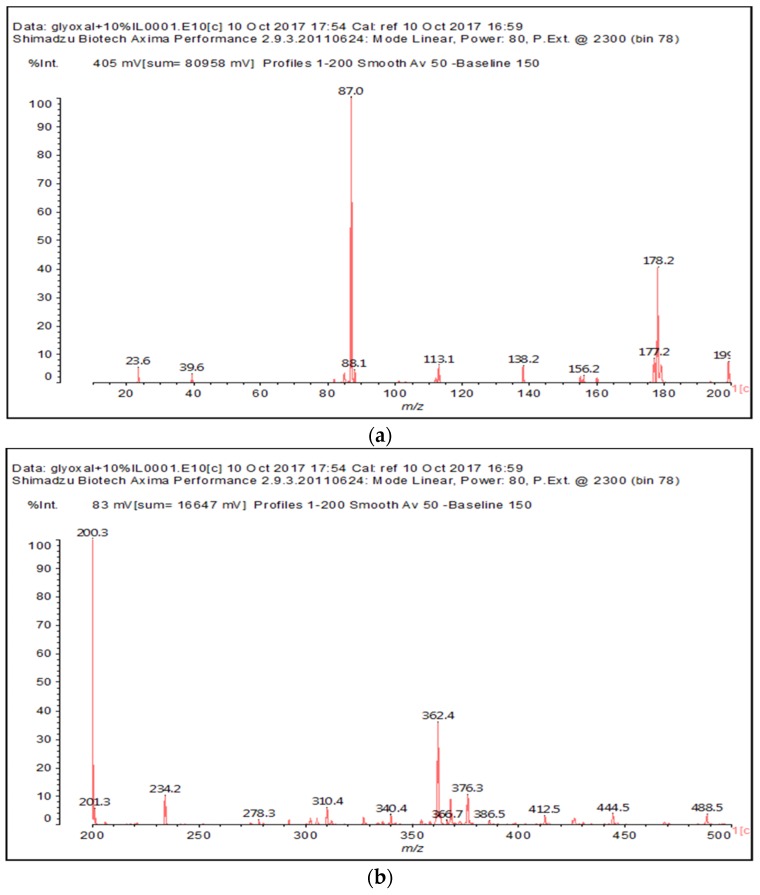
MALDI-ToF spectrum of the interaction between glyoxal and *N*-methyl-2-pyrrolydone hydrogen sulphate ([HNMP] [HSO_4_^−^]) ionic liquid: (**a**) 20–200 Da range; (**b**) 200–500 Da range.

**Figure 4:**
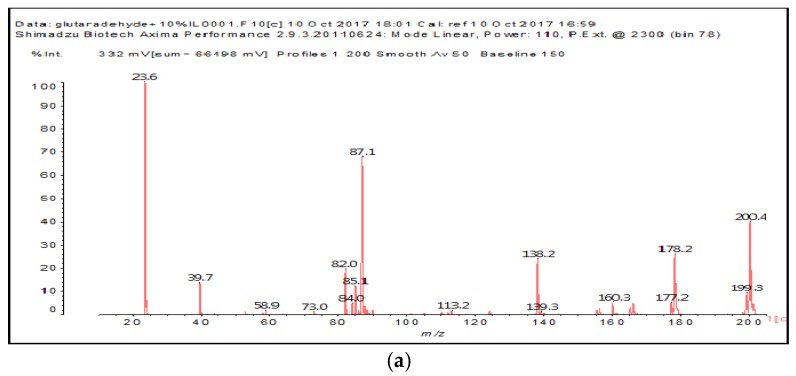

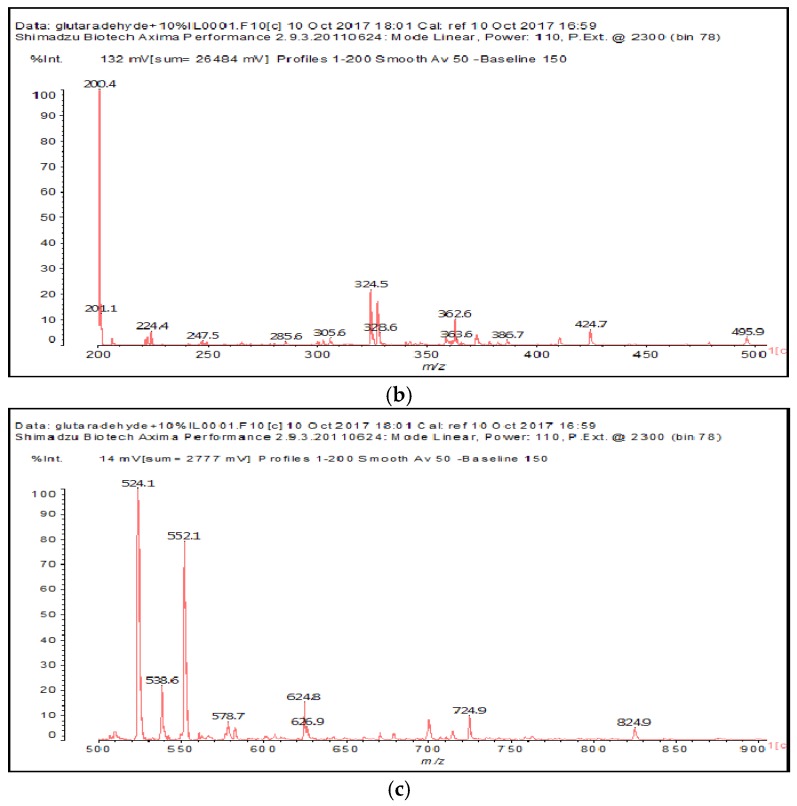
MALDI-ToF spectrum of the interaction between glutaraldehyde and *N*-methyl-2-pyrrolydone hydrogen sulphate ([HNMP] [HSO_4_^−^]) ionic liquid: (**a**) 20–200 Da range. (**b**) 200–500 Da range. (**c**) 500–900 Da range.

The MALDI-ToF of the two aldehydes first shown is for just the MALDI spectra of glyoxal and glutaraldehyde after reaction with the ionic liquid (Figure 3a,b and 4a–c). The species identified are shown in Table 3 and Table 4. Two main trends are noticeable:

Two main trends are noticeable, as shown below.

**Scheme 2:**
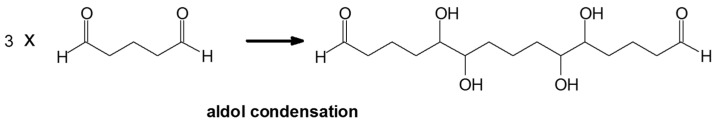
Example of glutaraldehyde aldol condensation.

**Scheme 3:**
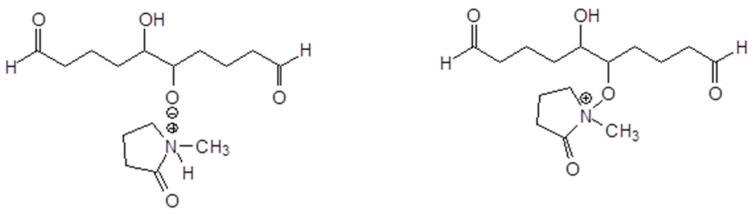
Examples of complexes between aldol condensates and ionic liquid.

1. Linear oligomerization of the aldehyde by aldol condensation, which indicates that IL has some catalytic effect on the autocondensation of the aldehyde (Scheme 2).
2. Formation of complexes between the aldol-derived oligomers formed with either *N*-methyl pyrrolydone or [HSO_4_^−^], or both (Scheme 3).

**Figure 5:**
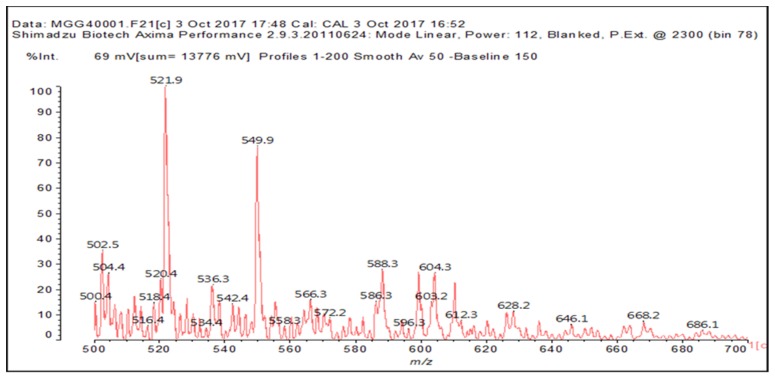
MALDI-ToF spectrum of the interaction between melamine and *N*-methyl-2-pyrrolydone hydrogen sulphate ([HNMP] [HSO_4_^−^]) ionic liquid. 500–700 Da range.

Figure 5 and Table 5 show the MALDI-ToF of the interaction between melamine and the ionic liquid. Only three peaks could be interpreted, namely the melamine peak, the ionic liquid peak and that of a coordination compound between two ionic liquid molecules and one single melamine at 536–539 Da (Table 4). The rest are fragments either coming from impurities or fragments generated in the MALDI. It is clear that the melamine itself is not affected by the IL, although species like that of 536–539 Da could facilitate reaction with the aldehydes.

**Scheme 4:**
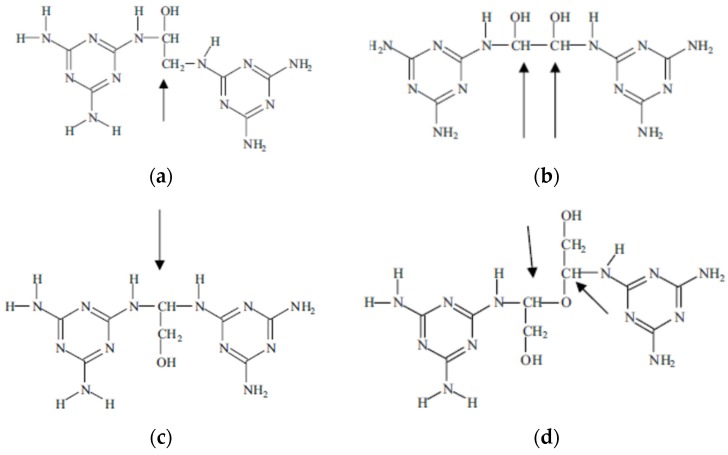
Type of bridges formed in MG resins.

In the case of MG resins several types of glyoxal bridges have been shown to form and link melamine molecules [13], such as the following (Scheme 4).

**Scheme 5:**
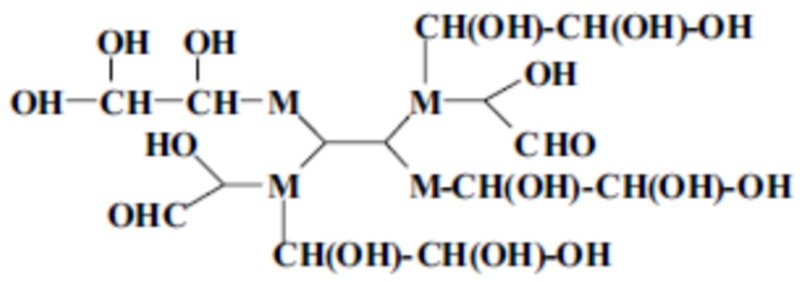
Type of branched oligomers formed in MG resins.

They also form and link very branched resins of structure of the type below (Deng et al. 2017) (Scheme 5).

**Figure 6:**
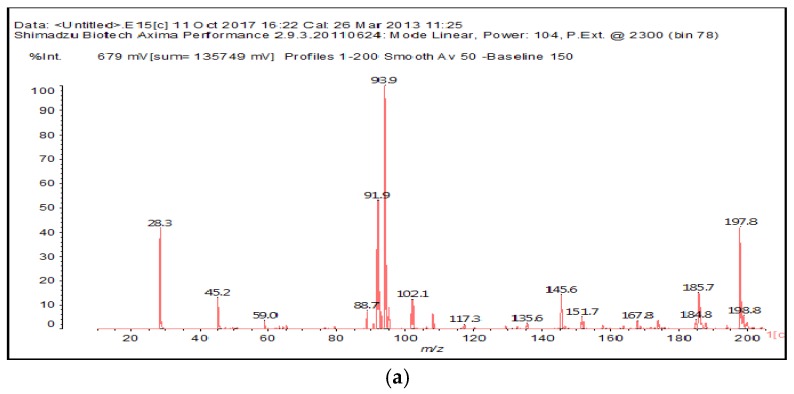

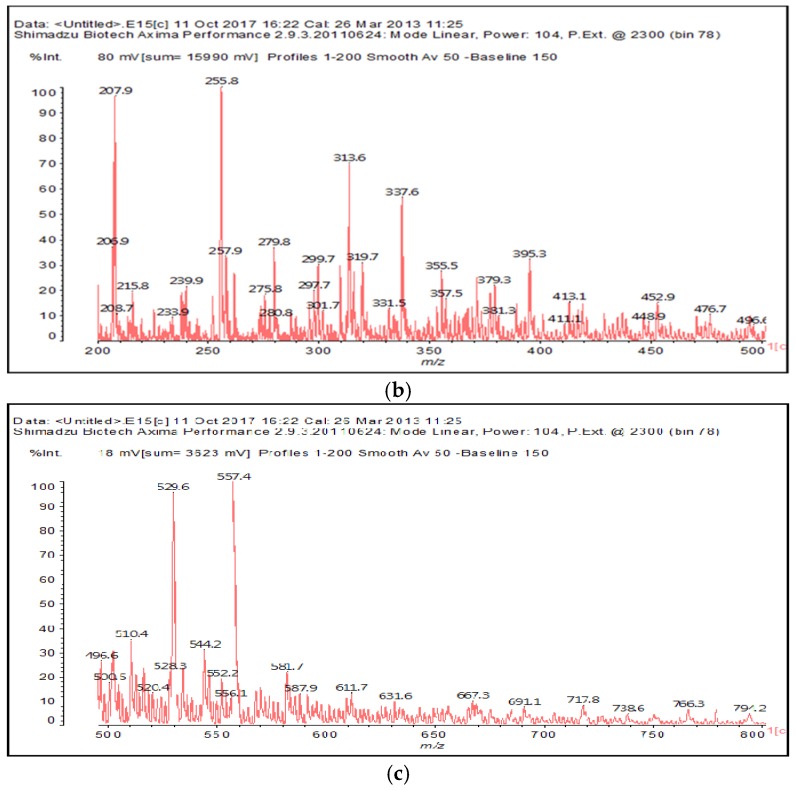
MALDI-ToF spectrum of the formation of the MGG’ resin in the presence of *N*-methyl-2-pyrrolydone hydrogen sulphate ([HNMP] [HSO_4_^−^]) ionic liquid: (**a**) 20–200 Da range. (**b**) 200–500 Da range. (**c**) 500–800 Da range.

Table 6 and Figure 6a–c, show what occurs when the resin MGG’ resin is formed when IL is added.

**Scheme 6:**
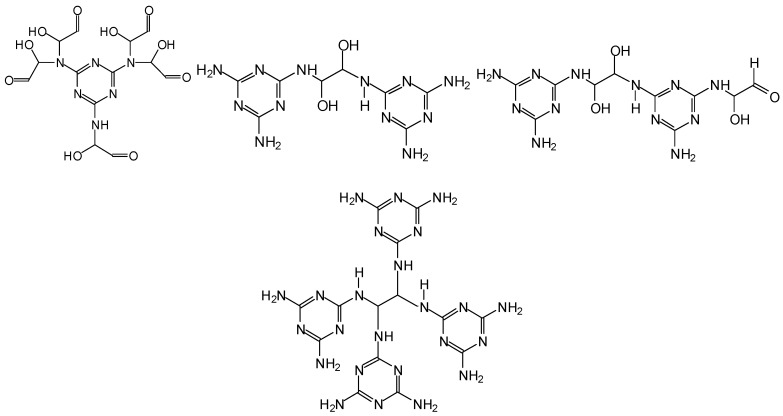
Oligomers types formed in MG resins.

In the case of the IL being present aldol condensation of the aldehyde occurs and species in which melamine is linked to aldol condensed aldehydes occur. For both the resin MGG’ with and without IL condensation oligomers of melamine–glyoxal and of melamine–glyoxal–glutaraldehyde are present. Thus species such as the following are the result (Scheme 6).

**Scheme 7:**
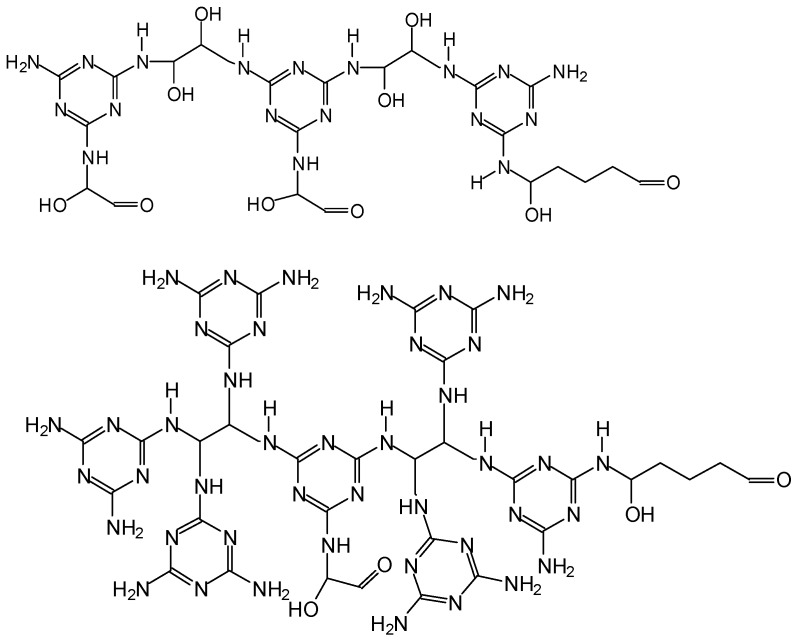
Oligomers formed in MGG’ resins showing reaction of glutaraldehyde and glyoxal.

Due exclusively to the condensation of melamine and glyoxal these species are present together with species on which glutaraldehyde is definitely grafted on. Some of these are (Scheme 7):

Where a glutaraldehyde is linked to a melamine of a MG resin skeleton.

**Scheme 8:**
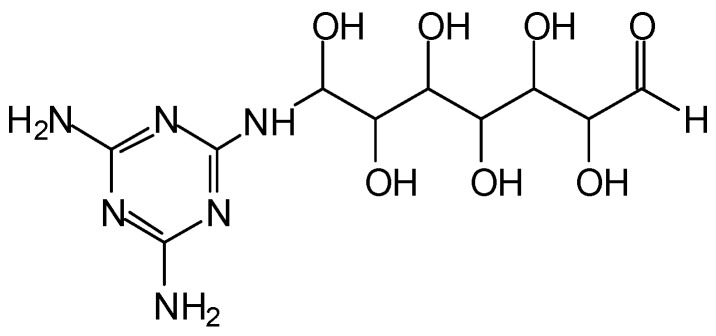
Reaction of a glyoxal aldol condensate on melamine.

Also species in which an aldehyde that has undergone aldol condensation has reacted and linked with the melamine are present in the MGG’ + IL resins. For example the peak at 356–357 Da (333 + 23 Da of Na^+^) in Figure 6 represent just one of these species issued by the aldol condensation of three glyoxals linked to a melamine molecule. Aldehydes react readily with melamine, but when a dialdehyde reacts after one of the two aldehyde groups has reacted and before the second one reacts aldol condensation can occur involving this second, still free aldehyde group. Thus either (i) the glyoxal units have condensed by aldol condensation for subsequently the residual aldehyde group to react with melamine; or (ii) one glyoxal molecule has reacted with melamine and the other glyoxals have then reacted on the residual aldehyde function by aldol condensation (Scheme 8).

**Scheme 9:**
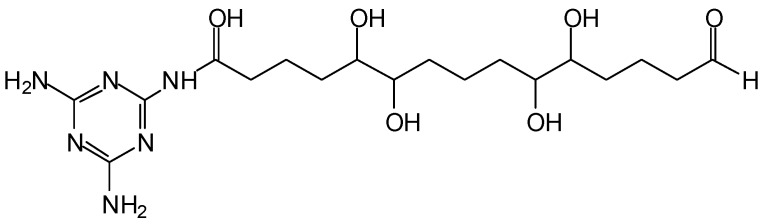
Reaction of a glutaraldehyde aldol condensate on melamine.

This is not the only case occurring in the MGG’ + IL resin case, as for example the 452–453 Da peak in Figure 6, this being a species formed by aldol condensation of three glutaraldehydes linked to a melamine (Scheme 9).

### 3.3. Analysis of Reactions by Fourier Transform Infrared (FTIR) Spectrometry

**Figure 7:**
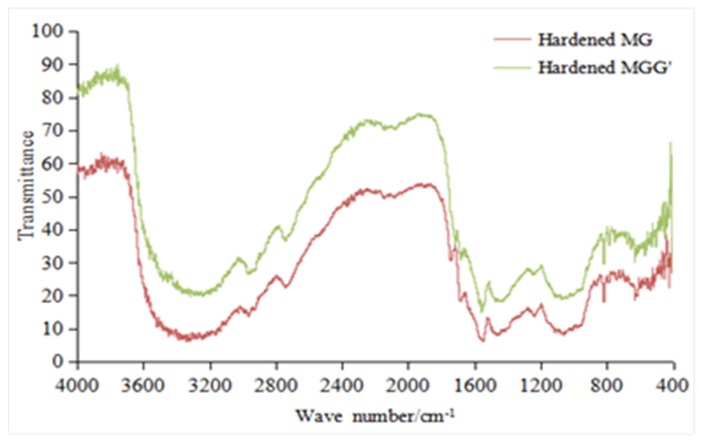
FT-IR spectra of hardened MG and MGG’ resins.

FTIR analysis of the hardened MG and MGG’ resins (Figure 7 and Table 7) shows all the main groups of the melamine and reacted glyoxal. These are listed in Table 6. The more interesting feature is the existence of the peak at 1234 cm^−1^ of C–O–C stretching. Its presence indicates that substituted methylene ether-like bridges (–CHR–O–CHR–) are formed in the case of MG resins just as they form in melamine–formaldehyde resins (MF, –CH2–O–CH2–). These substituted methylene ether-like bridges (–CHR–O–CHR–) clearly rearrange to substituted ethylene bridges (–CHR–CHR–) by the elimination of water as already shown in a previous study [13].

**Figure 8:**
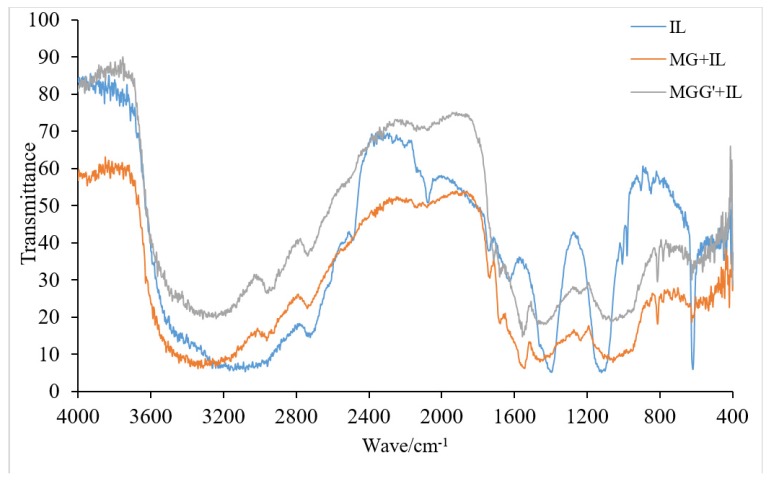
FTIR spectra of IL, MG + IL resin and MGG’ + IL resin.

**Scheme 10:**
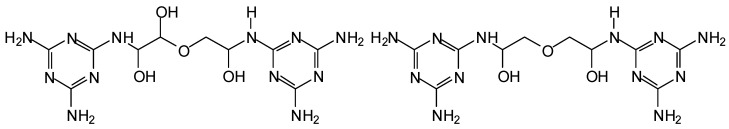
Glyoxal ether bridges formed by water elimination between two melamine molecules.

In Figure 8 there is a comparison of the FTIR spectra of IL, MG + IL and MGG’ + IL. Other than small peaks corresponding to the addition of the small amounts of IL the two resin spectra show the same peaks as shown in Figure 7. The probable presence of ethers in the structure is supported also by the existence of MALDI-ToF peaks such as those at 395 Da (371 + 23 Da Na^+^) and at 355 Da (without Na^+^) and 379 Da (with Na^+^) in Figure 6 which correspond to the two structures below (Scheme 10).

### 3.4. Summary of Effects

Having identified some of the more relevant of the oligomers that were found and shown that melamine and glyoxal do react and form resins which also cross-link it is of interest to summarize what is then the role of ionic liquids.

**Scheme 11:**
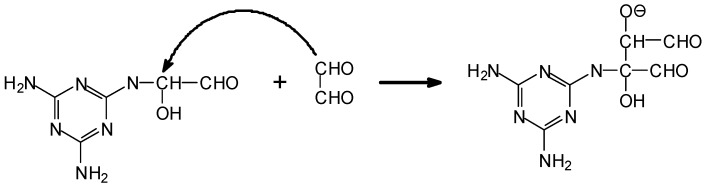
Example of aldol condensation on an aldehyde prereacted with melamine.

**Scheme 12:**
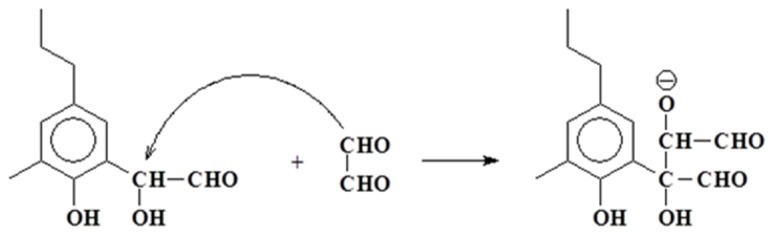
Example of aldol condensation on an aldehyde prereacted with lignin.

1. IL appears to catalyse the reaction of aldehydes, and aldehydes pre-reacted with urea or melamine, to yield aldol condensation. It allows aldol condensation even of aldehydes that have been pre-reacted with melamine or even with wood lignin. An example (Scheme 11):

Or even with wood lignin [22,23] (Scheme 12).

This latter opens up the possibility of reaction with the lignin in the wood substrate, and thus of some covalent bonding between an aldehyde based adhesive and the substrate. While to advance such a hypothesis is possibly premature, it might also contribute to explain the good bonds obtained with aldehyde adhesives catalysed by ionic liquids.

2. IL catalyses the hardening of melamine–aldehyde resins to decrease hardening temperature, energy of activation of the hardening/condensation reaction and thus equally to improve their performance at equal temperature.
3. Moreover, IL affects the wood substrate by demethylation of lignin [22,23,24] rendering the substrate even more receptive to any type of adhesion.

However, it cannot be excluded that also other effects are induced by the presence of ILs.

## 4. Conclusions

Melamine–glyoxal–glutaraldehyde (MGG’) resins which use no formaldehyde have been shown to be capable of bonding interior and exterior grade plywood under industrially significant press conditions when an ionic liquid is used as the hardener. Small proportions of glutaraldehyde improved the water resistance of the panel bonds obtained. These resins showed hardening in two steps due to the different reactivity of the two aldehydes used. The role of the ionic liquid was fundamental in the performance obtained, its role being not just limited to the function of hardener but also: (i) to decrease markedly the energy of activation of hardening of the MGG’ resins; (ii) to favour some limited aldol condensation of the aldehydes in the hardening of the MGG’ resins, even on aldehyde sites pre-reacted with melamine; (iii) to cause demethylation of lignin and thus rendering the wood substrate more prone to adhesion [23,24]; and (iv) to favour possibly the reaction of aldehyde groups in the resin with the lignin of the substrate.

## Figures and Tables

**Table 1:** Proportions of melamine (M), glyoxal (G) and glutaraldehyde (G’) used in the preparation of the MGG’ resins.

Resin Type	Relative Mixing Molar Ratio Glyoxal:Glutaraldehyde	Melamine (g)	Glyoxal (40% Solution) (g)	Glutaraldehyde (50% Solution) (g)
MG	100:0	25.2	174	0
MGG’1	95:5	25.2	163.5	12
MGG’2	90:10	25.2	156.6	24
MGG’3	85:15	25.2	147.9	36
MGG’4	80:20	25.2	139.2	48
MGG’4	75:25	25.2	130.5	60

**Table 2:** Plywood results for melamine-glyoxal-glutaradehyde (MGG’) resins. MG resin of mole ratio M/G = 1:6, pH = 4.5, reacted at 60 °C for 1 h. Three layers plywood were prepared and tested for tensile shear strength. Ten per cent by weight [HNMP] [HSO_4_^−^] ionic liquid was used as hardener.

Name of Resin	Relative Mixing Mole Ratio of Glyoxal:Glutaraldehyde	Solid Content/%	Viscosity/mPa·s	Dry Shear Strength/MPa	24 h Cold Water Shear Strength/MPa	2 h Boiling Water Shear Strength/MPa
MG	100:0	49	390	0.6	0.63	--
MGG’1	95:5	48.9	410	0.6	0.9	--
MGG’2	90:10	48.8	540	0.59	1.02	--
MGG’3	85:15	48.7	1720	0.76	1.39	0.45
MGG’4	80:20	48.5	2890	0.73	1.48	0.7
MGG’4	75:25	solidified in the reactor				

“--” means the samples broke.

**Table 3:** Species formed by the reaction of glyoxal with IL, by MALDI.

Peak	Chemical Species
199–200 Da = calculated 197 Da, proton = 198 Da	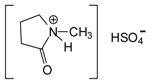
138 Da = 140 Da, glyoxal dimer by aldol condensation	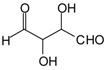
By aldol condensation as:	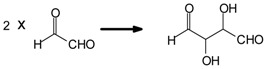
234 Da = 230 Da calculated, plus protonated, by aldol condensation, with Na^+^	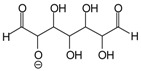
366–367 Da = 366 Da calculated, without Na^+^	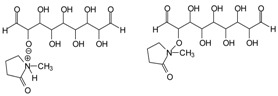
387 Da = 366 Da + 23 Da = as above but with Na^+^487–488 Da = with Na^+^	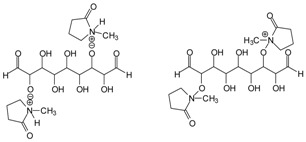

**Table 4:** Species formed by the reaction of glutaraldehyde with IL, by MALDI.

Peak	Chemical Species
199–200 Da = calculated 197 Da, proton = 198 Da without Na^+^	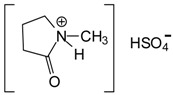
222 Da = as 199–200 but with Na^+^, however it is also possible 224 Da = 225 Da calculated with Na^+^ obtained by aldol condensation	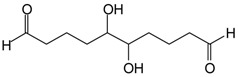
328.6 Da = 304 Da + 23 = 327 Da calculated, glutaraldehyde trimer by aldol condensation with Na^+^	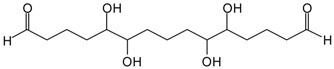
by aldol condensation, thus as	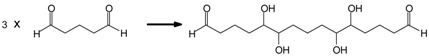
but also at 325 Da (observed 324 Da) thus more probable:	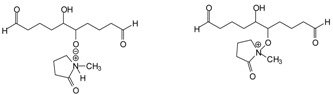
as well as coupling with [HSO_4_^−^] (see example of the 424 Da peak). 424 Da = 426 calculated, glutaraldehyde tetramer by aldol condensation with Na^+^but also	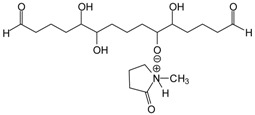
and also	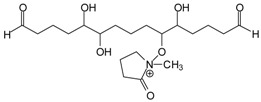
but also protonated + [HSO_4_^−^] such as	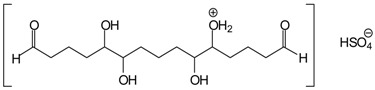
524 Da = glutaraldehyde pentamer by aldolcondensation, with Na^+^ but also the alternatives as for the 424 Da peak	---
624 Da = glutaraldehyde hexamer by aldolcondensation, with Na^+^ but also the alternatives as for the 424 Da peak	---
724 Da = glutaraldehyde heptamer by aldolcondensation, with Na^+^ but also the alternatives as for the 424 Da peak	---

**Table 5:** Species formed by reaction of IL with melamine alone. Only two peaks could be interpreted. The rest are fragments either coming from impurities or fragments generated in the MALDI. It is clear that the melamine itself is not affected by the IL, although species like the 538 Da could facilitate reaction with the aldehydes.

Peak	Chemical Species
200 Da = [HNMP] [HSO_4_^−^], calculated 197 Da, + protonation. 536–539 Da = 539 Da calculated (516 + Na^+^ = 539)	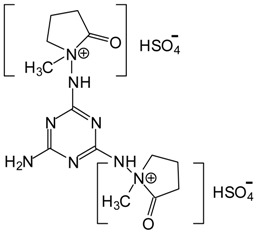

**Table 6:** Oligomers formed by the reaction of MGG’ with and without IL.

Peak	Chemical Species
149–151 Da = Melamine + 23 Da (Na^+^) with and without IL 159 Da = G + G’ by aldol condensation with and without IL. Small with IL 184–185 Da = M(Glyox)_1_ without Na^+^ in MGG’ with IL 197.8 Da = [HNMP] [HSO_4_^−^] (only present in MGG’ with IL) 234 Da = aldol condensation of glyoxal + Na^+^ exists only in MGG’ + IL	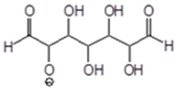
331.5 Da = 328.6 Da glutaraldehyde+IL by aldol condensation occurs but is relatively small in MGG’ + IL	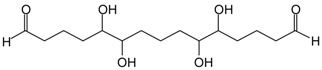
But also 331.5 = 310 + Na^+^ (333 calculated deprotonated = 332) in MGG’ + IL	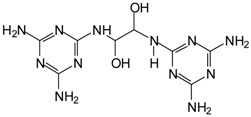
395 Da = 392 Da (369 + 23) calculated. MGG’ + IL with Na^+^	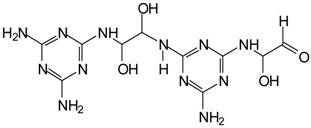
439 Da = MG5 + Na^+^, present in only MGG’	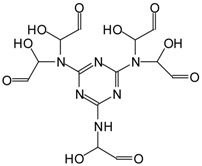
496 Da = 491 Da (with Na^+^), probably multiprotoated, in MGG’ + IL (= GMGMG’)	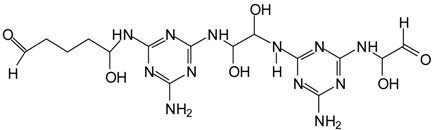
521 Da = 516/518 calculated = MG_5_G’ without Na^+^ or MG_5_G’ without Na^+^ of MGG’ + IL 536–539 Da = MG_5_G’ + Na^+^	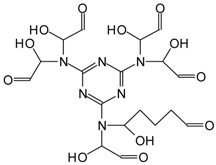
557.4 Da = 558 Da calculated = MG_4_G_2_’ of MGG’ + IL without Na^+^ 566 Da without Na^+^, in MGG’ 581.7 Da = 581 calculated = MG_4_G_2_’ with Na^+^ in MGG’ + IL 588 Da with Na^+^. 588 Da in both MGG’ and MGG’ + IL	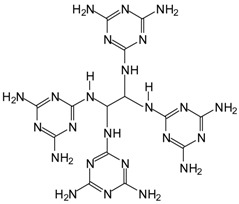
736–738 Da = 738 deprotonated, without Na^+^ In both MGG’ and MGG”+IL	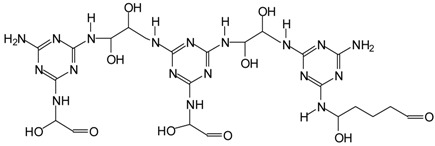
795–796 Da = without Na^+^ in both MGG’ and MGG’ + IL	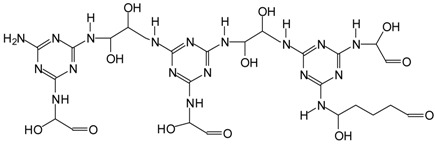
993 Da = 992 Calculated + protonated with Na^+^, only in MGG’	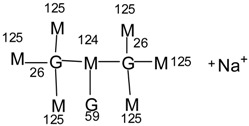
1010 Da = 1008 Da calculated + protonated, only in MGG’	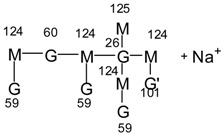
1169 Da = 1168 Da calculated, Without Na^+^	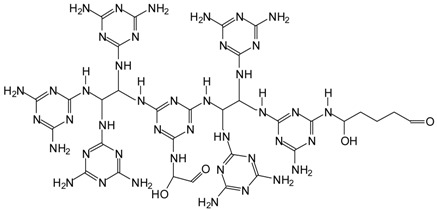

**Table 7:** FTIR spectra of hardened MG and MGG’ resins.

Wavelength/cm^−1^	Group
812	triazine bending vibration
1074.3	C–O stretching vibration
1235	C–O–C (ether group) stretching vibration
1460.1	Methylene C–H bending vibration
1543	Triazine C=N bending vibration
1550.8	Triazine C=N bending vibration
1678	C=O(carbonyl) stretching vibration
1735.9	C=O(ketone) stretching vibration
2739	C–H stretching vibration
2962.7	C–H(aldehyde) stretching vibration
3100–3200	N–H, O–H stretching vibration
